# Quantification of the Influence of Charge Variations on the Flow Behavior of Sheet Molding Compounds

**DOI:** 10.3390/polym16162351

**Published:** 2024-08-20

**Authors:** Anna Julia Imbsweiler, Junyan Wang, Reem Sharwalla, Julius Hoffelner, David Colin, Swen Zaremba, Klaus Drechsler

**Affiliations:** Chair of Carbon Composites, TUM School of Engineering and Design, Technical University of Munich, Boltzmannstr. 15, 85748 Garching, Germany

**Keywords:** SMC rheology, long fiber reinforcement, viscosity characterization, SMC charge configurations

## Abstract

Using a newly developed flow test bench, several charge configurations were analyzed to quantify the influence of the charge configuration in the mold in sheet molding compound (SMC) manufacturing. A test bench was developed to satisfy the industrial needs for the incoming goods inspection as well as the need for the flow characterization of rheological models in the simulation. The test setup has a cylindrical opening for the charge placement, from where the material is pressed into a thin flow channel, forcing the material to reorient. A comparison was performed by juxtaposing the resulting compression pressure recorded during the process. The charge for this test series, placed into the cylindrical opening, has two basal configurations, one consisting of a stack of disks, and the second in a rectangular sheet rolled up into a spiral. Six charge variations were tested in total. The amount of material, the batch, the layering and the production direction of the sheet proved to have a significant influence on the necessary compression pressure. Guidelines about the recommended charge configurations could be derived for optimized production settings, such as a reduction in the compression pressure and modifications to the charge.

## 1. Introduction

Weight saving has consistently remained one of the main goals in most industrial sectors over the past decades, as it reduces the necessary energy for any movement, consequently reducing the consumption of fuel or increasing the range of motion, saving money and emissions. Carbon fiber-reinforced plastic (CFRP) is commonly the material of choice for lightweight designs due to its high specific stiffness and strength. Short cycle times of only a few minutes, simple material handling and the possibility to create complex shapes have made sheet molding compounds (SMCs) a very common choice among CFRPs, especially in the automotive industry as a substitute for heavier metallic parts.

SMCs consist of two production steps, first producing the sheets and then compressing them into parts. In the first step, endless fiber bundles are chopped into long fibers of typically 25 mm in length that are randomly dispersed on a carrier foil coated in a resin layer. Another foil with a resin layer is added on top and compressed to obtain a fully impregnated sheet of around 1.5 to 2 m in width. The sheets typically display a thickness of 1 to 3 mm and the characteristics are assumed to be isotropic in the plane of the sheet. To manufacture a part with an SMC, the sheet is cut into a convenient shape, stacked to obtain the required mass of the final part and placed in a heated mold mounted in a press. Under a specific temperature, pressure and closing speed, the material is compressed and cured, flowing into every cavity of the geometry.

With typically 25 mm long carbon fibers, SMCs can offer high specific strength that, united with the processing experience of several decades, allows its adoption in high-performance applications [1,2,3]. The mechanical properties depend on the fiber orientation and the defects present in the final part. One of the causes for the final fiber orientation and the defects is the flow inside of the mold. This causes the fibers to reorient from a state in the raw material assumed as isotropic in the plane of the sheet [4,5,6]. It also induces most defects present in an SMC product, such as weld lines or short shots [7,8,9]. Therefore, it is imperative to understand the in-cavity flow and to quantitatively study the material and processing parameters affecting the flow behavior. 

The main material parameters include the fiber length, the fiber volume fraction, the fiber orientation and the tow size, where the main processing parameters are the mold temperature and the strain rates [10,11,12,13,14,15,16,17,18]. Furthermore, research has shown that, due to the movement of the carrier foil during the sheet production, a slight preferred orientation of the fibers in the direction of the movement of the foil is caused, which may influence the flow behavior of the material during the compression process [19]. The flow behavior also varies depending on the position over the thickness of the charge. More specifically, close to the mold, a lubricating layer is formed that flows faster than the material in the center, with the first, faster flow being referred to as the fountain flow, and the second flow referred to as the plug flow [20]. 

Due to this multitude of influences, SMC part producers have set several oral guidelines in order to ensure complete filling, good repeatability for final parts and sufficient mechanical properties. These guidelines include rough predictions of the necessary wall thickness to ensure complete filling, but also the charge shape and placement within the mold, the part orientation within the tool and the processing parameters. Examples include the closing speed usually being between 1 and 3 mm/s and the compression pressure being between 100 and 150 bar. Furthermore, in traditional SMC part production, the sheets are cut into a simple shape, like a rectangle, and stacked until the desired weight of the final part is reached. This stack is placed inside of the preheated mold in a central and flat location. During the compression, the material is compacted significantly until reaching its yield stress and the void content is reduced to a minimum, before it flows into the entire cavity. Typically, the flow length is kept small, cutting the material to a shape close to the final part, covering between 50 and up to 80% of the mold. With a high coverage, most of the material does not experience significant flow, leading to the final part having the isotropic mechanical properties of the uncured SMC. These mechanical properties are typically higher than the performance obtained in parts experiencing long flow lengths. Furthermore, a near-net shape increases the probability of the complete filling of a part. However, with a near-net shape, the amount of scrap from the sheet cutting increases. Moreover, ribs are a very common reinforcement. To ensure complete filling, and to reduce the likelihood of resin-rich areas and the formation of weld lines on the tip of the rib, additional SMC pieces are placed inside of the ribs before the compression. This process is highly inefficient, since an SMC sheet has to be cut separately and each piece has to be placed inside of the designated rib, needing much more time than the placement of one sheet stack. Furthermore, when producing an SMC part, the adopted material is the same in terms of the chemical composition for all units. However, if one batch finishes, a new batch of the same material is used, and it cannot be exactly known how far the curing of a specific material has progressed. Resin and hardener are mixed when the sheet is produced and the pot life at room temperature is usually 1–1.5 months. Typically, the material is stored at very low temperatures, increasing the pot life significantly. Nevertheless, the curing progresses continuously, not allowing for a clear assessment of its progression before using the material in the production. It is unknown whether and how much the change in batches or the curing progression influences the processing. 

Thus, with a better understanding of the influence of the charge and its possible variations on the process and the implications for the mechanical performance, SMC parts could become thinner, and the charge configuration as well as the production settings can be optimized. Within this study, six charge configurations are derived from the challenges presented and are tested. The resulting compression pressure is compared. The comparison is accompanied by a simple visual inspection of the specimen tips in relation to the original charge shape and its deformation to adapt to the cavity. A complex 3D flow pattern is necessary in order to reflect the real parts, such as a formula student car rim [3] or a trunk lid [21]. 

The traditional flow testing setup for SMCs is squeeze flow, consisting of two parallel plates. These are mounted in a universal testing machine (UTM) or in a press for SMC processing. The plates may be equipped with pressure or temperature sensors to track the temperature development and the pressure profiles [14,16,17,18,20,22,23,24,25,26,27,28,29,30,31,32,33,34,35,36,37,38,39,40,41]. However, the measurement only considers in-plane flow, as the sheets are placed parallel to the plates and can only flow more or less freely in the plane of the sheets. This reflects the real placement of the charge with the stacked sheets. However, this does not reflect the more complex flow pattern in a 3D shape similar to a real SMC part. In order to depict a more complex planar flow with forced changes in the orientation of the fibers and a high degree of interaction with mold surfaces, as well as a long flow, a spiral flow test was introduced by Rabinovich et al. [42]. The setup consisted of a curved channel equipped with sensors. The material flowed for a considerable distance, being multiples of the charge length. Nevertheless, the flow of the material happened only parallel to the sheet plane. 

Another class of flow test setups comprises more complex testing tools with out-of-plane flow to be used either in a press or in a UTM and equipped with pressure and temperature sensors to track the temperature and pressure in different locations on the part and the flow speed [43,44,45,46]. However, Dumont et al. [43] do not provide insight into complex structures like ribs. Kim et al. [46] do display a rib structure, but the application is for a UTM. For the ideal transferability of the results to a typical SMC process, an application in a typical SMC press shall be preferred. The reason is the limited closing speed provided in a UTM, as well as the different closing speed variation in the dependency of the applied force. The thermal management can also be expected to be different, since the mass of the heated material in a press exceeds the heated material in a UTM, usually limited to the compression plates. Therefore, in the latter configuration, a much faster dissipation when opening the tool and a less uniform distribution of the heat can be expected overall. Nevertheless, the significance these differences have on the flow behavior of the material have not been investigated. Rothenhäusler et al. [44] and Hohberg et al. [45] investigated the same geometry, displaying structures similar to a rib. However, the adopted tool did not provide an accurate tracking of the overall compression pressure, since it relied on the data provided by the press. Moreover, the included pressure sensors in the abovementioned solutions were aimed at tracking the local pressure inside of the mold in a specific smaller area and the flow front overall. 

A third option for SMC flow testing is an independent test stand [47]. The mold consists of a bar equipped with temperature sensors along the flow channel. The mold offers the possibility to select among three different thicknesses for the channel. This setup fulfills the requirements for the complexity and the vicinity to a rib geometry. However, as the pressure is applied through a pneumatic actuator, it can only apply the rather low pressure of 9 bar, which is far from around 100–150 bar, the typical pressure in an SMC compression step [48]. Furthermore, the pressure and temperature profiles are quite different from an application in a press.

Therefore, a newly developed bar flow test bench is used here to investigate and quantify the influence of charge variations on the compression pressure in order to improve the current understanding of the flow behavior. 

## 2. The Flow Test Bench

The newly developed test bench adopted for this study has been developed for the following two separate goals:

- Obtain data for the calibration of SMC material cards for flow simulations;
- Understand the influence of different charge variations on the flow behavior of an SMC in a complex shape, common in SMC parts.

For the first goal, in order to perform the process simulations of the SMCs, several aspects have to be characterized. The most important parameters to describe the flow of the SMCs are those for the curing kinetics of a thermosetting compound and the viscosity. These are determined with differential scanning calorimetry and squeeze flow tests, respectively. However, squeeze flow tests only consider a very simple flow configuration, presenting no flow obstacles that impede the flow. Therefore, it is not sufficient to reflect actual contemporary SMC parts. Hence, several SMC part producers have stated that they avoid running SMC process simulations, since the results are not reliable. To improve the results, the material card is calibrated. This calibration consists of producing an SMC part in a press using the mold of a geometry similar to the one to be simulated. The process was numerically replicated and the two results were compared. The parameters of the curing kinetics or of the viscosity were iteratively adapted until the empiric pressure signals coincided with the numerical ones. This process was necessary, since the simple flow depicted in squeeze flow tests was not sufficient to reflect the complex flow present in the SMC parts, especially not in simulations exhibiting several simplifications of the material description. At the same time, increasing the level of detail of the simulations was not possible with current computational capacities because the material and its interactions during the flow were too complex to be accurately modeled. No standardization exists for this process, preventing any comparison between the results of the parameter adaptation for the same material. Moreover, the process is tedious, with no guarantee that the identified material parameter set is suitable to depict the flow in a different geometry. 

**Figure 1:**
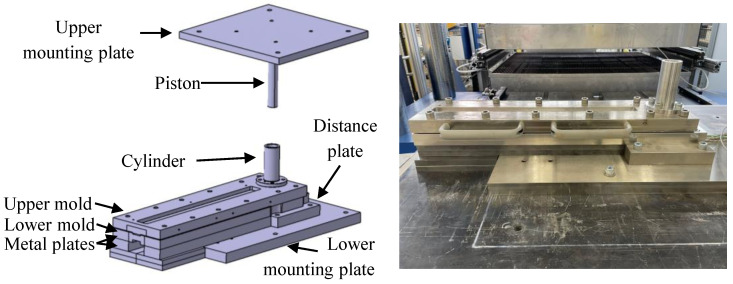
Setup of the test bench and identification of the main components.

The second aim of the test bench is the comparison of different charge variations on the flow behavior and processing of the material when producing 3D complex shapes. As stated in the Introduction, a suitable solution for a test bench, satisfying all requirements, does not exist yet. Therefore, a new flow test bench was developed that can recreate a more complex flow state and typical industrial processing parameters for SMC parts, as shown in Figure 1. 

The test bench consists of an upper half mounted to the top plate of a press with a mounting plate. The main feature of the upper half is the piston, which will apply the pressure on the SMC during the test. The lower half consists of the main mold, which is completely closed. Furthermore, a cylinder is placed at the beginning of the mold to hold the raw material that is going to be compressed. The overall length of the setup is about 0.5 m, while the exact measurements of the flow channel are specified below in Figure 4. 

**Figure 2:**
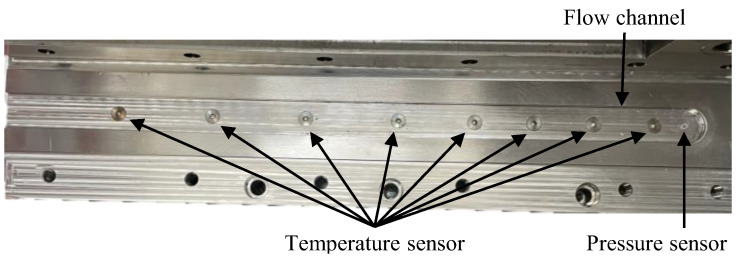
Inside of the lower mold with a pressure sensor at the beginning of the flow channel and eight temperature sensors aligned along the 450 mm long flow channel.

Along the lower mold, several in-mold sensors were placed to track the temperature and pressure data, as shown in Figure 2. 

Right underneath the piston, there is a cavity pressure sensor, of the type 6163AC3,0 from Kistler Instrumente GmbH (Sindelfingen, Germany), to record the compression pressure. A load amplifier from Kistler Instrumente GmbH (Sindelfingen, Germany), a measuring amplifier, the QuantumX MX410B (Hottinger Brüel & Kjær GmbH, Darmstadt, Germany), and the software MX Assistant 4 (Kistler Instrumente GmbH, Sindelfingen, Germany) were used for the data acquisition. The sensor can be calibrated to 100 or 1000 bar. Since the geometry is more complex than traditional SMC parts, the expected pressure values resulting during the compression were expected to be in the range of 100 to 300 bar, partly slightly higher than the typical average. The values were closer to 100 bar, which was defined as the reference for the calibration. Along the flow channel, eight additional temperature sensors were placed to track the temperature of the channel. The temperature data also allowed for a quantification of the flow speed, as the temperature signal was disturbed once the material reached a sensor. Furthermore, the exothermal curing reaction could be identified due to a noticeable temperature increase. The first four temperature sensors were placed closer together for a more precise flow front tracking at the beginning of the compression. Additionally, the in-mold sensors can track the overall temperature of the setup. For the same purpose, three temperature sensors were placed on top of the upper mold, one on the cylinder and one on the piston.

**Figure 3:**
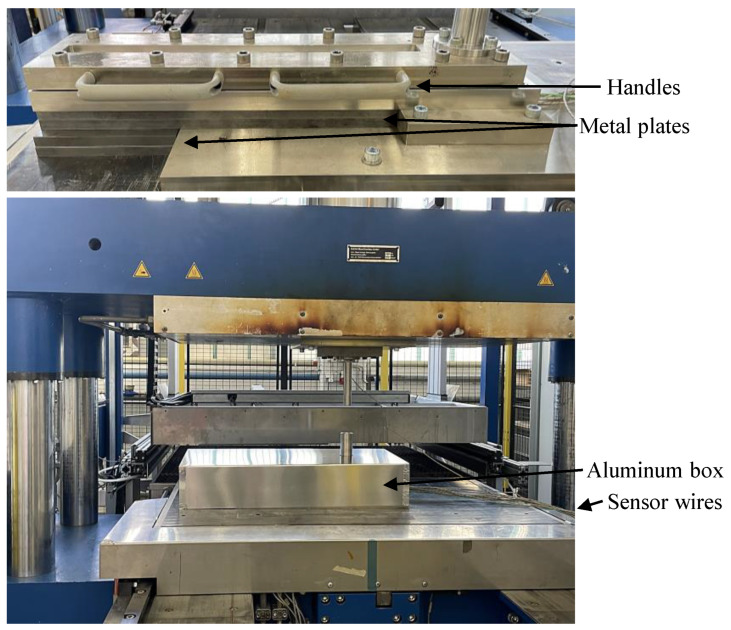
Bar flow test setup inside of the RUCKS thermoforming press covered with an aluminum box for improved thermal management.

Figure 3 shows the sensor wires from the lower mold exiting the setup on the left. 

The test bench was developed to be adopted in a RUCKS Thermoforming KV 289.00 machine (RUCKS Maschinenbau GmbH, Glauchau, Germany), which can apply the typical processing conditions present in SMC part manufacturing. Those include a heated upper and lower plate, a constant closing speed during the compression and the application of the required pressure. Several metal plates were introduced to hold the mold, improving the stability, and to spread the temperature from the heated press more evenly. Moreover, an aluminum box was built to closely fit over the setup. To extract the specimen after the test, the mold has to be opened. For improved usability, handles were attached to the upper mold to make the opening more convenient.

**Figure 4:**
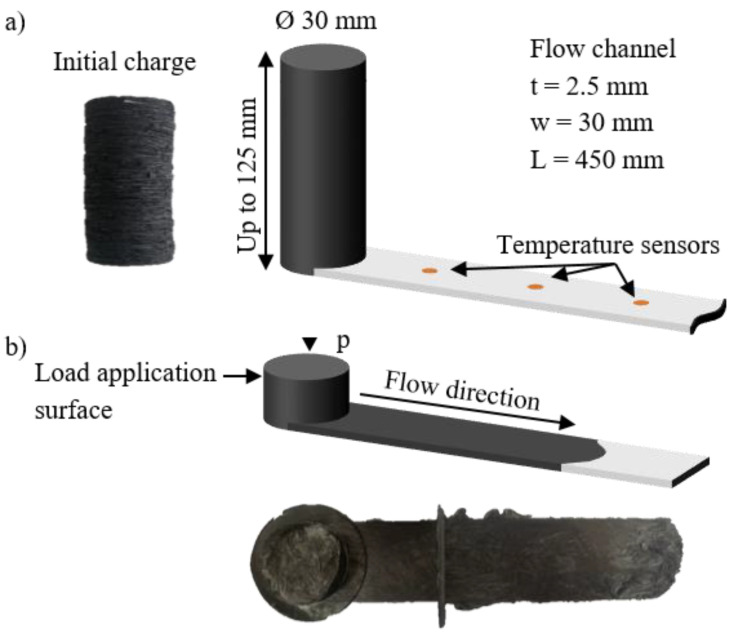
Functioning principle: (**a**) measurements of the cavity and the initial charge, with exemplary charge from the test series; (**b**) load application surface and flow direction with exemplary specimen from the test series.

The general functioning principle of the test bench, with the closed mold and the moving piston, is shown in Figure 4. 

A cylindrical charge was placed inside of the cylinder, and the piston pressed it into the bar inside of the closed mold. Once the compression was complete, the press stayed closed for up to 10 min to ensure the full curing of the material. After the opening of the press, the specimen can be taken out of the closed mold by unscrewing the upper mold half from the lower mold half. The opening of the mold reduces the overall temperature of the setup, which has to be reheated to the desired temperature before performing the next test. The cavity consists of a 450 mm long, 5 mm thick and 30 mm wide flow channel, as shown in Figure 4a. The thickness of 5 mm exceeds a typical rib thickness, which is typically around 3 mm. However, normally, the ribs in the SMCs are also not as long as the flow channel in this case. Therefore, a wider thickness was chosen for this first version of the test setup. The width of the channel was chosen to be 30 mm in order to be as small as possible, but still a little wider than the fiber length to facilitate the flow in the central area and to have at least a small amount of fibers not redirected by the walls or cut for the charge preparation. The overall geometry of the cavity was intended to reflect a challenging flow case to be completed by the material, representing the opposite end of the complexity spectrum compared to the squeeze flow tests performed for the material characterization. Within the investigation of the charge variations, this allows for the differences in the flow behavior to be more evident. Furthermore, for the simulation-related goal, this shall allow for a quantification of the error resulting from simulating the bar flow setup with the parameters obtained directly from the squeeze flow tests. However, the evaluation of the simulation-related goal of the test bench will be included in a future study, since the study at hand focuses on the second aim of the test bench, the investigation of the charge variations. The length of 450 mm is relatively long for an SMC in order to be able to cover the cases that exceed the typical applications. 

The developed bar flow test bench has several significant advantages, which are as follows:

- Allows for a complex flow, given the high degree of reorientation and deformation happening for the charge in order to enter into the flow channel through a small opening;
- A closed shape compared to a squeeze flow setup, comparable to out-of-plane structures present in real SMC parts in a closed mold;
- A structure comparable to ribs, a very common feature;
- The use in a production press, allowing for the use of industrially relevant pressures and closing speeds;
- A constant compression speed is applicable, as in the industrial process;
- Good thermal management;
- Good tracking of the compression pressure;
- Good transferability of the setup into simulation for the calibration of material parameters;
- The flexibility of the setup, with the modular sizing of the molds and additional plates;
- Good maintainability due to easy exchange of relevant parts, such as the contact surface area in the upper mold.

Nevertheless, the setup presents a few challenges that shall be addressed in the future, among which include the tracking of the exact position and the closing speed. A feasible solution is the installation of a displacement sensor. The press tracks the displacement, but a specific device would increase the precision. Furthermore, a future variation could be performing the flow tests under vacuum, since several SMC part manufacturers apply vacuums inside of the mold during the compression. Nevertheless, for the purpose of this study, the flow test bench is suitable as presented above.

## 3. Specimen Configurations and Material

**Figure 5:**
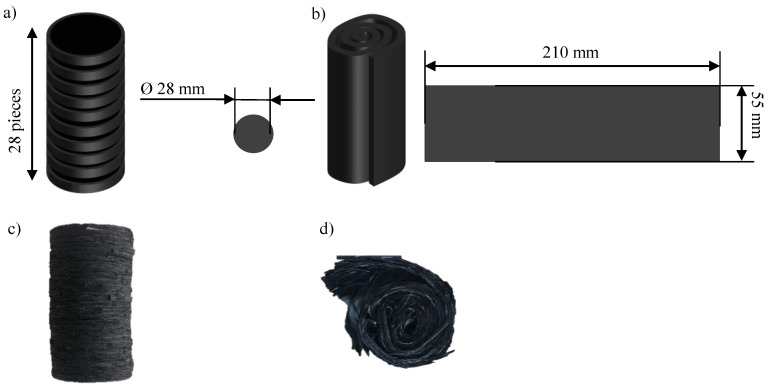
The charge configurations considered in this study: (**a**) configuration of a disk pile charge; (**b**) configuration of a spiral charge, consisting of a rolled-up rectangle; (**c**) prepared disk pile charge; (**d**) prepared spiral charge.

The material adopted in the study is CARBKID PGK5250-R63 (Astar S.A., Zangroiz, Spain) [49]. The material presents a fiber weight content of 52%, a 25 mm fiber length, 3 k tows and is based on a vinyl ester resin. The following two charge configurations were considered as a basis in this study (as depicted in Figure 5): a disk pile and a spiral charge. 

As can be seen in Figure 5a, 28 disks of a diameter of 28 mm comprised one disk pile 0charge. The spiral charge shown in Figure 5b consists of a rectangular sheet of 210 × 55 mm that was rolled up into a cylinder of approximately 28 mm in diameter. In Figure 5c, a disk pile charge specimen and, in Figure 5d, a spiral charge specimen can be seen before the test. As can be seen in the last picture, due to the aforementioned layering in the SMC production, when rolling up the spiral, the layers tend to separate from each other, creating additional waviness inside of the spiral. 

These two base configurations were further varied to a total of six test series (Table 1).

Each configuration was repeated five times in order to capture as much as possible of the spectrum of results due to the variability in the material, and also to be able to stipulate a statistically relevant conclusion. The aspects of the variability of the SMC relevant to the flow include many aspects, among which are the density, the actual fiber orientation and the thickness. The adopted material is commercially available and provided in a coil. In order to reflect normal industrial application, the specimens were cut over the entire width of the coil, only avoiding the most external 5 cm, as recommended by SMC part manufacturers.

The considered variations are related to the amount, the orientation and the age of the material. The amount variation consisted in using double the amount of material, meaning 56 disks stacked for the disk charge and two spirals stacked for the spiral charge. The definition of “oriented” in this case is related to the production direction of the sheet. As mentioned in the Introduction, the sheets are assumed to be isotropic in the plane. However, this is not true, as there is a slight preferred orientation of the fibers in the movement direction of the carrier foil [50]. Therefore, in the oriented charge, all of the disks were stacked so that the production direction and, consequently, the preferred orientation of the contained fibers was the same. In the “non-oriented” pile, the disks were alternated in their orientation to replicate an orthogonal stacking. The third variation is the moment in the pot life, since, in an industrial context due to transportation, handling, storing temperatures, etc., the exact moment in the pot life of a material is not known. To this end, the material obtained four months prior to the tests was defined as “within pot life”, as it was stored following the requirements from the producer and, therefore, was compliant with all of the requirements for industrially adopted SMCs. A second batch, defined as “after end of pot life”, had been in refrigeration for 13 months. According to the recommendations of the producer, this period exceeds the acceptable storage time, and curing should have progressed too much to still be used for production. Both batches were stored as recommended at −21 °C from the time of delivery. The reference configuration is the non-oriented single-amount disk pile charge with new material, as this replicates the most common charge configuration in SMC manufacturing. The variations were applied based on this configuration.

## 4. Process Conditions

**Figure 6:**
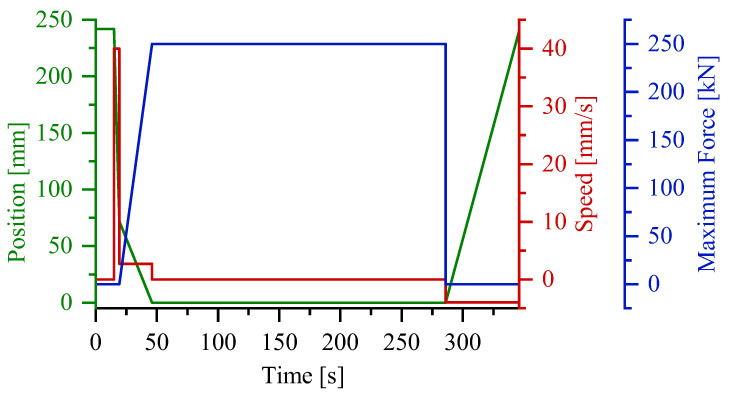
Press profile showing the upper tool displacement position and speed, and the maximum force set for the compression step.

To guarantee the applicability of the obtained results to a series production of SMCs, the compression process is displacement-controlled. The process profiles of the piston displacement, the force and the closing speed are shown in Figure 6. 

After inserting the specimen, a timer of 15 s was started, after which the press program was started. The recording of both the pressure signal and the temperature signal were started at the same time. Before the contact with the material, the piston moved at a speed of 40 mm/s, before slowing down to 2.7 mm/s. The compression speed was set as a percentage of the possible compression speed. For the performed flow tests, it was set to 40% of the compression speed, which was recorded to be 2.7 mm/s. This aligns with an industrial SMC process, where the closing speed is set in a range between 1 and 3 mm/s. The maximum force to be held once the compression was completed was set to 250 kN. Once the press was closed, a timer of 4 min was started to allow for the material to completely cure before reopening the press. The temperature was set to 160 °C, with a tolerance of ±5 °C for the sensors inside of the flow channel. Once the press was open, the specimen was extracted from the mold. Due to the mold opening and the contact with air at room temperature, the mold cooled down rather fast. Therefore, before performing the next test, the setup was heated back up to 160 °C.

## 5. Results

**Figure 7:**
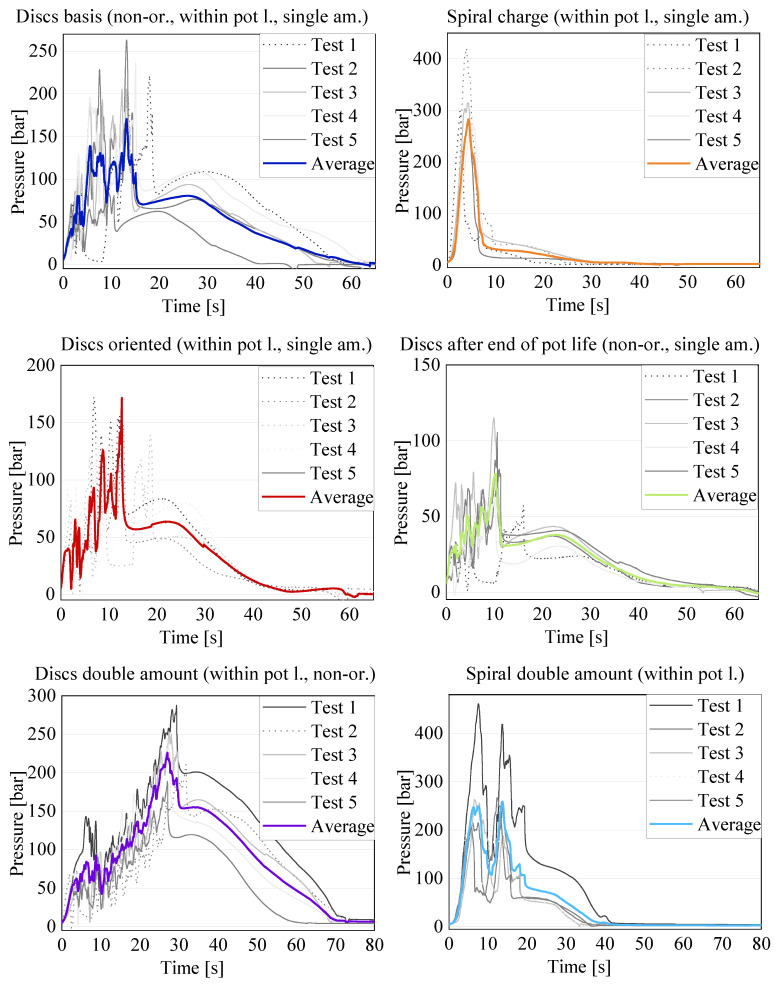
Results for all of the performed tests and the averages calculated for each sequence.

In a first step, a qualitative comparison of all of the results obtained for each test setting was performed. To this end, the results of all five tests for each configuration and the related averages can be seen in Figure 7. A direct comparison between the different settings and quantitative considerations will follow afterwards. 

**Figure 8:**
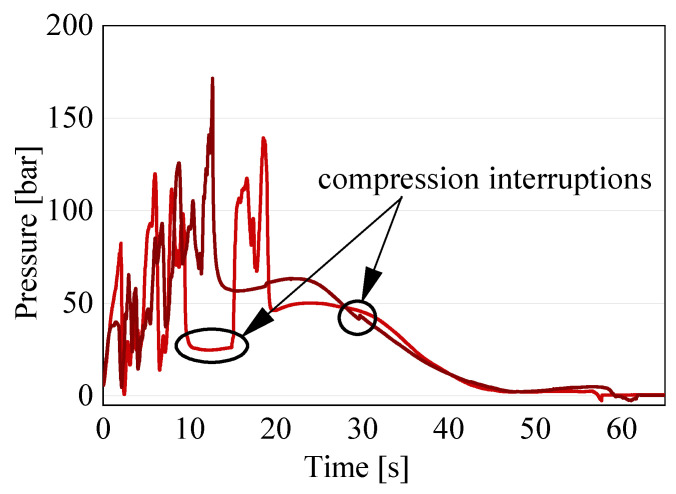
Exemplary interruptions during the compression reflected in the recorded pressure signal (tests 3 and 5 from the series with the oriented charge).

The start of the compression varied among the specimens for the following two reasons: the charges had varying heights, which varied the time of the first contact, and the light barrier of the press was oversensitive and sometimes interrupted the compression. Due to these two factors, the first contact with the material could vary by 2 to 3 s. Therefore, in order to calculate the averages, the moment the pressure recorded by the sensor underneath the piston was 5 bar was set to t = 0. This adaptation allowed for a better comparison of the pressure signals, which are the relevant result. The compression was completed after about 50–60 s, which is observable in the results due to a drop in the pressure signal after the highest peak. Afterwards, a slight expansion of the material occurred during the curing, leading to a slight and steady increase in the pressure signal, before dropping to almost zero for the remainder of the process. From the test signals, it is evident that, for each configuration, the results of the pressure signal are variate, as expected due to the variability of the material. Furthermore, all of the disk charges showed an oscillating signal with several pressure increases and drops, whereas the spiral specimens showed an overall linear development. All of the configurations showed an increase in the pressure signal until the peak. The increasing force necessary to press the material into the bar-shaped channel caused the peak. The increase in the compression force was caused by the cross-section normal to the movement direction of the material, becoming smaller from the cylinder to the channel, and, therefore, increasing the amount of material to be moved. The compression end criteria, were either a force maximum of 250 kN or the piston end position in the cylinder was reached. The latter corresponds to a final material thickness underneath the piston of 2 mm. In all of the tests, the final position was reached, meaning that the material reached complete curing during the compression in none of the tests. Only a start in the curing could be confirmed for several tests by a slight increase in the temperature of 1–2 °C, as recorded by the temperature sensors in contact with the material, which would also contribute to an increase in the compression force. Once the compression stopped, the pressure dropped as the material relaxed for a very short time before slightly increasing again during the curing, where the material expanded slightly. It is visible that, even for the tests with the same parameters and charges, the pressure peaks differed in value and in occurrence time. Both aspects were due to the varying heights of the specimens. However, this is not the only cause for the differences, as explained in more detail below. For the calculation of the averages, only the continued lines were chosen. The dotted lines in Figure 7 are the result of tests where the compression was interrupted due to the oversensitive photoelectric barrier of the press. The change in the signal due to such an interruption can be seen in Figure 8, explaining the reason for the exclusion of these curves from the average. 

The interruption of the compression led to a relaxation of the material and, consequently, a significant decrease in the pressure signal. The error was quit within seconds, as can be seen in Figure 8, but the peak pressure was not valid for a comparison. For the test series with the oriented disks, unfortunately, every test except for one had interruptions during the compression. When the interruption happened after the peak, as exemplarily shown for the dark red line in Figure 8, the test was considered for the average, as the topic of interest is the compression step.

**Figure 9:**
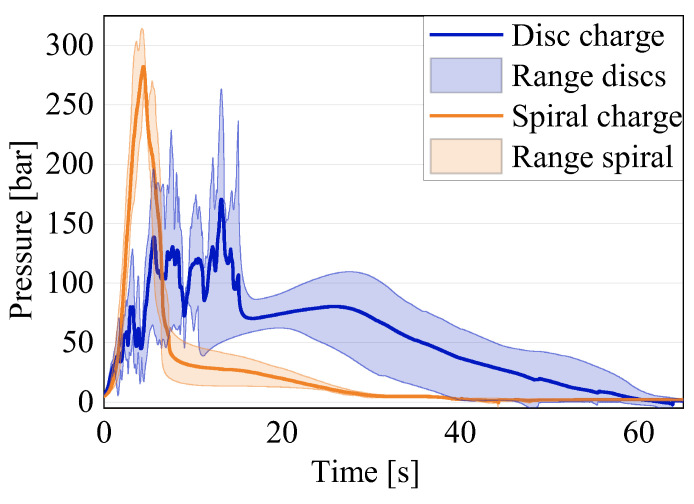
Comparison between the single-amount non-oriented disk pile charge and the spiral charge.

When considering the resulting pressure curves more in detail, in a direct comparison between the results for the disk pile charge and the spiral charge the pressure signals show clear differences (see Figure 9).

**Figure 10:**
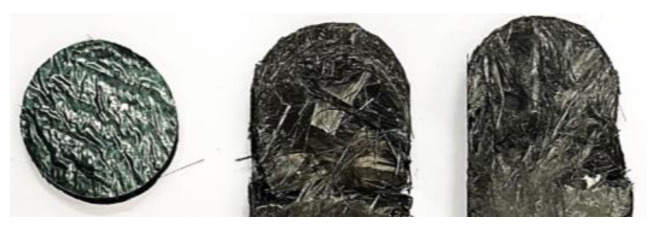
Disk pile specimen tips displaying the round shape of the initial disks next to one disk of an uncured SMC (with carrier foil), as used for the charge pile for the size comparison.

The average for the basis (single-amount disk pile charge, with non-oriented disks and new material), represented in blue, shows a clear oscillation, which can be explained with the specimen configuration. At the beginning of the flow, the fibers are lying in the flow plane, and the cutting edges of the disks are open towards the channel, which makes it easier for the material to flow. During the compression, a small lubricated area is formed between the sheets, leading to the disks being squeezed into the flow channel separately or in small batches. This leads to the oscillating signal, since, at the beginning, there is a linear pressure increase while the material collects in the cylinder. When the pressure is high enough, there is a sudden release in the pressure as one or a few disks pour into the flow channel, favored by the lubricating layer formed between the sheets. This process repeats itself until the compression stops. This behavior can be seen in all of the specimens with a disk pile charge, and is confirmed by all of the specimens where, at the tip of each specimen, the disk shape is still clearly distinguishable, as can be seen in Figure 10.

**Figure 11:**
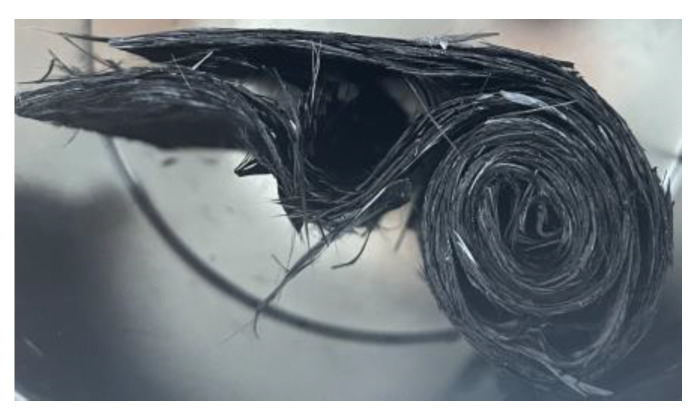
Spiral charge showing the recurring separation between inner and outer layers of fibers within the sheet.

The rise and drop of the pressure signal repeats itself until all of the material is in the flow channel or the curing has already started and the viscosity has become too high for the material to continue flowing. For the spiral charge, the pressure increase is linear with a very steep slope. This is due to the orientation of the sheet in the charge, as the fibers are oriented in a plane perpendicular to the flow plane, meaning that the fibers have to reorient before being able to flow into the channel. The configuration of the uncured sheet is even more complex due to the separation between sheet layers when rolling up the specimen. This phenomenon can be seen in Figure 11. 

This complex material configuration hinders the flow significantly. Thus, the flow can only be achieved by applying a high pressure. Furthermore, the spiral charge is one piece, with no interruptions in the flow direction, leading to the linear increase in the pressure signal. An overview of the relevant data for the comparison can be seen in Table 2. 

The maximum pressure reached during the tests for the disk charge varied between 84.69 bar and 263.20 bar. The lowest pressure corresponded to the lowest charge weight of 49.5 g. However, the highest weight tested, being 52.3 g in test 3, only reached 205.61 bar, the second-lowest value. Therefore, against previous expectations, a higher amount of material does not necessarily lead to an increase in the peak pressure. The spiral charge had an average peak pressure of 282.31 bar, higher than the highest peak pressure obtained for the disk pile charge. The high pressure was due to the complex configuration of the charge, as previously shown in Figure 11, and the fiber orientation perpendicular to the flow direction. Therefore, the orientation and shape of the charge is of the utmost importance, as it can double the necessary compression pressure. When producing complex parts, the charge should be placed so that the cutting edges are facing the longest flow direction in order to guarantee complete filling and to keep the compression pressure low. Structures like ribs perpendicular to the sheet are reflected in the test with the spiral charge, allowing us to conclude that the necessary pressure for successful filling has to be considerably higher. 

**Figure 12:**
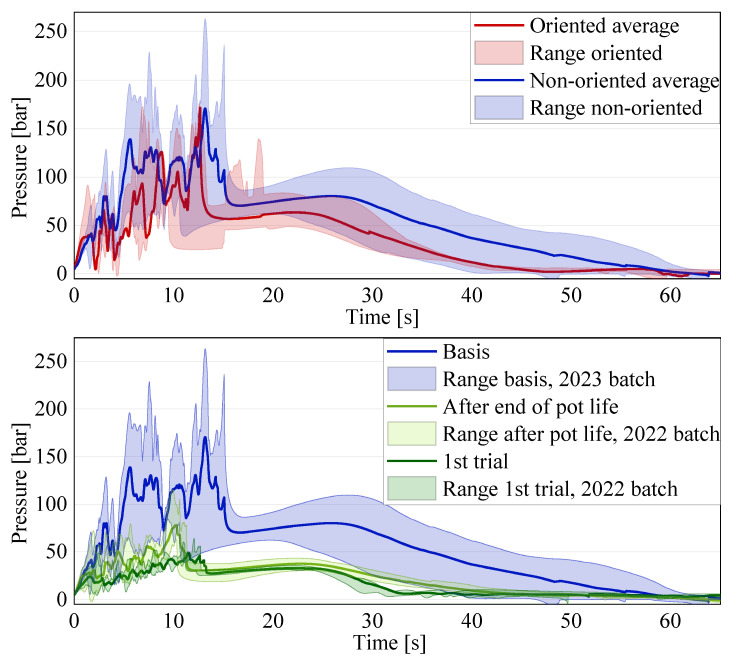
Results for disk pile charges compared to the reference (in blue): (**top**) oriented (red) vs. non-oriented reference (blue); (**bottom**) old material newly tested (light green) vs. old material tested one year earlier (dark green) vs. reference with new material (blue).

Another, more detailed, comparison has also been carried out between the basis (single-amount disk pile charge with no preferred orientation) and the results for the variations in the single-amount disk pile charge, as can be seen in Figure 12. 

In all of the curves for the average, the aforementioned oscillation of the pressure signal can be seen. It is consistent for all of the tests performed with the disk pile charges. The first diagram shows the comparison between the reference with non-oriented disks and the pile charge with all of the disks oriented in the same direction as the flow channel. The range for the oriented charges includes all performed tests, whether or not they had an interruption during the compression. It is visible that the highest peak in the oriented charge is 171.53 bar, which is only 0.9 bar higher than the peak in the average curve for the basis (exact data are shown in Table 3). However, the range of the non-oriented charge includes several peaks exceeding the average significantly. Thus, an undoubted conclusion about the influence of the orientation on the necessary compression pressure is not possible at this time due to the weight differences in the charges and the lack of results for the opposite orientation option for a final validation. However, the slight preferential orientation of the fibers in the sheet production direction seems to have an influence on the necessary compression force when producing an SMC part, since the flow of the material is better in the fiber direction. This means that the stacking orientation of a charge of an SMC is very important in order to reduce the production costs, as the compression pressure can be reduced significantly. When the processing settings remain the same but the charge orientation varies, as it happens in current SMC production with symmetrical charges, the flow of the charge will be different and, therefore, the final parts will differ. In order to quantify the influence of the orientation fully, an additional evaluation with an oriented pile, placing the sheet production direction perpendicular to the flow direction, is planned for the future.

Table 3 shows the key values for this first and the following comparison. 

**Figure 13:**
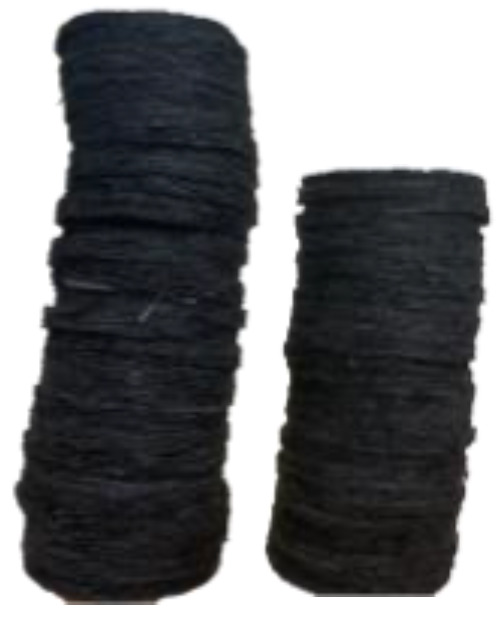
Two disk pile charges, with 28 disks with different heights due to varying sheet thicknesses due to the different positions in the coil.

Another interesting result can be seen for the comparison between the tests with respect to within or after the pot life, as well as the difference between the batches of the same material, as shown in Figure 12 (bottom). The pressure signals for the tests performed with the old material, despite being one year apart, are very similar: the peak pressure for the new tests is 78.01 bar, and 49.40 bar for the tests performed one year prior. According to SMC producers, the material can be conserved at −21 °C for about a year in order to still be processed without problems. Afterwards, the curing has already proceeded enough for the material to be partially or fully hardened. However, the results show that the conservation of the material in the freezer does not lead to a significantly different compression pressure, even when stored over one year. Nevertheless, the results differ significantly from the reference, with the peak of the new tests being 45.7% of the reference peak. All of the specimens of the old material have a lower weight due to a prolonged contact with air and, therefore, an extended time for the volatile components, like styrene, to be set free, and the location at which the specimens were cut from the coil due to the thickness variations. The lower weight contributes partially to the difference in the pressure signal compared to the basis. However, the decrease should not be as significant when comparing the signal variations within one batch. Further investigations are necessary to explain the difference between the two material batches fully, as they are labeled as the same material. The authors are currently not aware of changes in the resin formulation. As can be seen in Table 3, the weights of the charges show a variation of up to ±2 g, which is due to the difference in the thickness of the industrially produced SMC sheets. This difference in thickness also leads to a difference in the specimen height and, therefore, the start of the compression, which corresponds to the first contact of the piston with the material. Figure 13 exemplifies this issue, showing two specimens before being tested consisting of 28 disks each with very different heights. 

**Figure 14:**
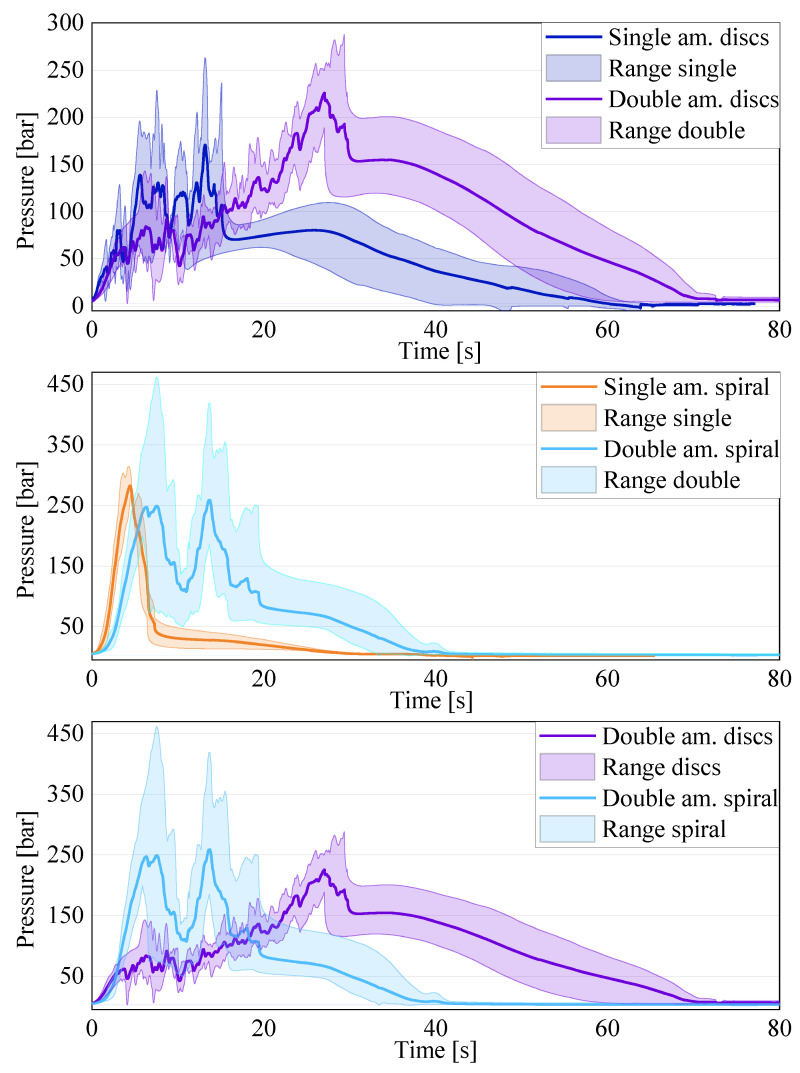
Comparison of the averages of the results for the double-amount charges with single amounts.

Therefore, in a future evaluation, it could be interesting to perform the tests with the same weight for the charge, independent of the amount of disks used. The same difference appeared in the tests for the double-amount charges, as shown in Figure 14. 

The upper diagram shows that an increase in the material amount leads to an increase in the overall pressure, with the average for the double amount being 225.92 bar compared to the 170.63 bar of the single amount. Nevertheless, the oscillation of the pressure signal, observed in all previous disk pile charge results, is also visible in this configuration. The results for the double-amount spiral charge are shown in the intermediate diagram. The linear increase in the pressure signal is visible for both the single and the double amounts. However, all of the results for the double-amount charge show a drop in the pressure signal at half of the displacement, followed by a second steep increase, which corresponds to the separation of the two single-amount charges constituting the double amount. However, it is clear that the variation in the results for the single amount is very low, while for the double amount it is quite significant. Therefore, if a rather predictable pressure development is desired during the process, a more complex charge configuration shall be preferred. For the double-amount spiral charge, the results vary significantly, leading to an average value of the pressure peak being lower than the pressure peak for the single amount. However, this does not apply to all of the performed tests. Therefore, when transferring the results to industrially relevant applications, such as to the filling of a rib, it is evident that the separation between sheets can decrease the pressure signal significantly. This is due to the material from the first charge entering the flow channel without any hindrance from fibers perpendicular to the flow direction. The second charge starts flowing into the channel only when the first one is already in the channel. Therefore, the separation between the two charges favors the flow speed. However, it also interrupts the homogeneous flow of the material, changing the flow pattern as described above. This means that, when a charge is placed perpendicular to a rib, a cut in the charge underneath or in close proximity to the rib will decrease the necessary compression force significantly and improve the probability of a complete filling with no weld lines in the rib tip. Nevertheless, the cut fibers will be shorter and, on the edges, a resin-rich area might be formed. In order to be able to confirm the deduced advantages for rib filling, a study shall be performed in the future. In order to fully understand and quantify the influence of the separation and the charge weight, additional tests shall be performed in the future in order to allow for quantified production guidelines. A summary of the key results for the above-shown diagrams for the single- and double-amount charges can be seen in Table 4.

The oscillation of the charge weight observed previously for the disk pile charges is also visible for the double-amount charges and for the spiral charges. In the double-amount disk charge, the weight variation for 56 disks increased to ±3.6 g. For the double-amount spiral charge, the variation in the weight was even at ±6.6 g. Furthermore, the heaviest spiral charges for both the single and double amounts caused the highest peak pressures of 420.05 bar for the single amount and 461.38 bar for the double amount, hinting at an overall weight dependence, which was already observed previously. However, the weight dependency cannot be confirmed for all of the specimens, since exceptions are also present. Therefore, further investigations into the relevance of the charge weight shall be performed in the future. Furthermore, in order to exclude the weight dependency of the results, tests shall also be performed for the basic configuration with all of the specimens having the same weight. 

In the direct comparison of both double-amount charges, the qualitative development of the curves is very similar to the single amount, with the peak values of the pressure being much more similar. Nevertheless, this highlights again the importance of the separation within the charge in changing the flow behavior and the compression pressure. Nevertheless, the microstructural consequences still have to be investigated in the future, since the different flow behavior surely has an influence on the structural properties, as well as the charge configuration by itself. 

**Figure 15:**
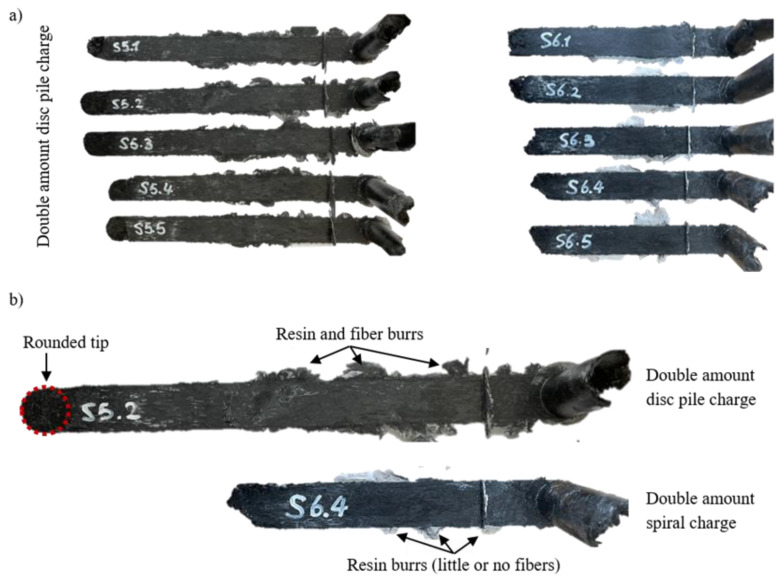
Specimens resulting for the double amounts: (**a**) all specimens for the double-amount disk and spiral charges; (**b**) comparison between a double-amount disk charge (**top**) and a double-amount spiral charge (**bottom**).

As Table 4 shows, the weights for the spiral charges compared to the disk charges are much lower. Figure 15 displays a direct comparison between the resulting charges. 

It is evident that all of the disk pile charges (Figure 15a on the left) have a significantly longer flow distance than the spiral charges (Figure 15a on the right) due to their weight difference. In the detailed comparison in Figure 15b, the rounded tip reproducing the contours of a disk is visible, confirming the repeatability of this behavior throughout all of the disk pile charges. Another visible difference between the specimens is the burrs. In the disk pile charge, the burrs contain a mixture of fibers and resin, whereas, in the spiral charge, they mainly consist of resin. This is due to the fiber orientation within the charge, which, in the spiral charge, leads to the close entanglement of the fibers. Therefore, they cannot easily separate from the bulk, i.e., when forming a burr. In the disk pile charge, on the other hand, the open edges in the disks and the flow direction corresponding with the orientation of the fibers is favorable to the fibers moving freely within the resin. This further confirms the necessity of open edges in the flow direction, as well as the avoidance of complex charge shapes, in order to achieve complete filling even in complex geometries.

The study has shown many influences on the resulting specimens, and especially on the resulting pressure signal. Especially in processing regards, being able to keep the compression pressure low decreases the production costs significantly. Therefore, the possibility to choose the charge accordingly represents a considerable benefit. A summary of the influence of the charge configuration on the peak pressure is shown in Table 5. 

It is visible that the basis configuration of the disk pile charge has comparable pressure values to the variation with the sheets oriented in the production direction. However, a definitive statement cannot be made in this regard, since only one curve for the oriented charge could be considered. Furthermore, the basis configuration has the highest peak in comparison with the remaining tests with single amounts. However, the material batch seems to have a more significant influence than whether the material is within or after the pot life, since all of the tests with the material batch bought one year prior resulted in significantly lower pressure values. Therefore, despite the compression pressure being significantly lower for the newer material in this study, it cannot be generally stated that an older batch leads to lower compression pressure. With regard to the pot life, since it is the recommendation of the producers not to process the material any more, the curing was expected to have progressed far enough to lead to very high compression pressures and very bad flow patterns, with only some areas of the sheet able to flow. The results have shown that, even significantly after the official pot life, the material displays the same compression pressure as the same batch within the official pot life. Nevertheless, it has to be mentioned again that the exact moment within the pot life can never be known for the material to be compressed. For the basic configuration and the variations in the single amount, it is clear that the necessary pressure is very similar for the same material batch, but each batch needs to be tested separately to determine the necessary pressure level. A significant reduction in the compression force can be obtained by changing the charge arrangement, since, in comparison with the spiral charge, the pressure for the disk pile charge is decisively lower. However, the development of the pressure signal is much more predictable, with the spiral charge displaying a very steep linear increase. This overall behavior of the spiral charge is also visible in the double-amount charges. For the disk pile charge, the double amount leads to a longer increase in the compression pressure and the related increases and drops, and an overall higher compression pressure. However, a direct proportionality between the material amount and the peak pressure could not be seen, since the peak pressure for the double amount was lower than double the value for the single amount. For the double amount of the spiral charge, the pressure was slightly lower than for the single amount, underlining the importance of the separation present in the charge that leads to a significant drop of the compression pressure. Nevertheless, the average value for the double-amount disk pile charge was still slightly lower than for the double-amount spiral charge. Therefore, a simpler charge configuration with the edges of the sheets open towards the main flow direction leads to the lowest compression pressure and is, therefore, recommendable if that is the desired goal.

## 6. Conclusions

All of the applied charge variations showed an influence on the outcome of the pressure signal, meaning that the charge configuration does have an influence that, in some cases, is even significant. It could be shown that all of the configurations of the disk pile have an oscillating pressure signal due to the layering in the charge and the formation of lubricating layers between the disks. It could be confirmed that the sheet production direction does contribute to the resulting pressure, as, with all disks oriented in the flow direction, the pressure signal was lower than in a charge that also included disks perpendicular to the flow. Furthermore, each material batch seemed to be different and, therefore, had to be tested to know the required compression force. However, this has to be further investigated in order to better understand the exact origin of the difference. Nevertheless, it could be concluded that storing the material at −21 °C for over one year does maintain the flow characteristics, leading to an almost unchanged pressure signal. The spiral charge proved to be the least convenient, as the resulting pressure was much higher than for the disk pile charge. This can be compared to the filling of a rib or a complex structure that is placed perpendicular to the flow direction, showing that the pressure necessary to fill such an area is much higher than if the part were less complex. When performing the tests with the double amounts, an overall direct proportionality between the amount of material and the pressure increase could be observed in the disk pile, although exceptions have been pointed out. For the double-amount spiral charge consisting of two single-amount spiral charges, a significant pressure drop can be seen when the separation between the two spirals reaches the channel entrance. Therefore, when producing complex structures like ribs that are perpendicular to the sheet surface, cutting the charge on the surface in proximity to the rib can greatly reduce the necessary pressure to fill the whole geometry. As described above, this observation also leads to speculation that a separation in the charge can increase the chances of a complete filling, reduce the probability for the occurrence of weld lines on top of the ribs and reduce the turbulences in the flow, minimizing fiber entanglement. However, due to the cut in the charge, it can also reduce the mechanical performance. In order to understand fully the transferability of these results to real complex SMC parts and ribs in particular, further investigations shall be performed in the future. These findings and the derived guidelines shall improve the definition of a charge in order to reduce the production costs, and to improve the repeatability and the probability of a quality part as an outcome. Nevertheless, several aspects have to be further investigated and quantified more precisely, such as the influence the disk orientation has, the applicability to other bar thicknesses and a wider channel, the influence of the charge weight as well as other configurations like a continuous double-amount spiral charge. Moreover, an approach has to be developed to include these configurations in filling simulations. For the developed flow test bench, the applicability for one of the two main goals of allowing for the investigation of charge variations has been shown within this study. The validity of the flow test bench for its second goal of performing the calibration for the material parameters of the curing kinetics and the viscosity for complex cavities will be part of a future study.

## Figures and Tables

**Table 1:** Performed charge variations.

	Disc		Spiral	
**Volume**	Single 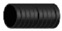	Double 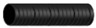	Single 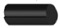	Double 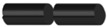
**Orientation**	Oriented 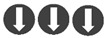	Non-oriented 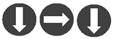	-	
**Age**	new	one year old	-	

**Table 2:** Key results for the basis disk charge (single-amount, quasi-isotropic, new material) and the single-amount spiral charge.

	No.	Peak Pressure [bar]	Weight [g]	Compression Start [s]
Disc charge	1	221.68	50.7	37.1
	2	263.20	51.4	36.2
	3	205.61	52.3	33.4
	4	236.29	50.4	32.8
	5	84.69	49.5	39.4
	average (2–5)	170.63	50.9	
Spiral charge	1	298.75	39.3	48.5
	2	420.05	44.5	36.8
	3	314.41	34.7	40.1
	4	271.32	33.9	46.0
	5	283.23	30.8	40.5
	average (3–5)	282.32	33.1	

**Table 3:** Key results for the basis, the oriented charge, the tests with the material that had exceeded pot life and for the old material in tests performed one year prior (weight not available).

	No.	Peak Pressure [bar]	Weight [g]	Compression Start [s]
Basis *	1	221.68	50.7	37.1
	2	263.20	51.4	36.2
	3	205.61	52.3	33.4
	4	236.29	50.4	32.8
	5	84.69	49.5	39.4
	average (2–5)	170.63	50.9	
Oriented	1	172.51	44.2	−
	2	147.97	45.9	39.7
	3	139.40	46.9	38.7
	4	175.79	46.7	33.2
	5	171.53	46.5	39.1
	average (5)	171.53	46.04	
After pot life	1	57.31	43.5	38.1
	2	81.11	44.2	39.9
	3	115.06	43.1	35.5
	4	62.64	41.7	40.8
	5	105.46	41.7	35.8
	average (2–5)	78.01	42.84	
1 year old tests	1	56.10	−	37.9
	2	56.28	−	39.0
	3	60.22	−	39.5
	4	63.97	−	38.2
	average (1–5)	49.40	−	

* quasi-isotropic, new material and tests.

**Table 4:** Key results for the single- and double-amount disk pile charges, and for the single- and double-amount spiral charges.

		No.	Peak Pressure [bar]	Weight [g]	Compression Start [s]
**Discs**	Single amount	1	221.68	50.7	37.1
		2	263.20	51.4	36.2
		3	205.61	52.3	33.4
		4	236.29	50.4	32.8
		5	84.69	49.5	39.4
		average (2–5)	170.63	50.9	
	Double amount	1	287.54	94.9	17.2
		2	210.65	92.9	19.4
		3	252.08	94.2	17.4
		4	233.68	89.5	23.3
		5	188.69	87.8	19.9
		average (1, 3–5)	225.92	91.6	
**Spiral**	Single amount	1	298.75	39.3	48.5
		2	420.05	44.5	36.8
		3	314.41	34.7	40.1
		4	271.32	33.9	46.0
		5	283.23	30.8	40.5
		average (3–5)	282.32	33.1	
	Double amount	1	461.38	74.6	27.7
		2	251.72	71	28.6
		3	262.81	67.6	32.6
		4	224.69	60	30.9
		5	266.65	61.4	28.2
		average (1–3, 5)	258.67	68.65	

**Table 5:** Summary of the influence of each charge configuration on the peak pressure comparing the left to the right.

		Oriented	After Pot Life	1 Year Old Tests	Double Amount	Single Amount	Double Amount
Discs	Basis *	↑↓	↑↑	↑↑	↓↓	↓↓	─
	After pot life			↑	─	─	─
	Double amount					─	↓
Spiral	Single amount						↑
					* single, non-oriented, new material		
	Peak Pressure						
	↑/↓	higher/lower					
	↑↑/↓↓	much higher/lower					
	↑↓	indifferent					

## Data Availability

The original contributions presented in the study are included in the article, further inquiries can be directed to the corresponding author.
